# Acoustic Wake-Up Technology for Microsystems: A Review

**DOI:** 10.3390/mi14010129

**Published:** 2023-01-03

**Authors:** Deng Yang, Jiahao Zhao

**Affiliations:** 1 Department of Precision Instrument, Tsinghua University, Beijing 100084, China; 2 Key Laboratory of Smart Microsystem (Tsinghua University) Ministry of Education, Tsinghua University, Beijing 100084, China; 3 State Key Laboratory of Precision Measurement Technology and Instruments, Tsinghua University, Beijing 100084, China; 4 Beijing Laboratory of Biomedical Detection Technology and Instrument, Beijing 100084, China; 5 Beijing Advanced Innovation Center for Integrated Circuits, Beijing 100084, China

**Keywords:** acoustic wake-up, microsystem, acoustic transducer, acoustic recognition, system architecture

## Abstract

Microsystems with capabilities of acoustic signal perception and recognition are widely used in unattended monitoring applications. In order to realize long-term and large-scale monitoring, microsystems with ultra-low power consumption are always required. Acoustic wake-up is one of the solutions to effectively reduce the power consumption of microsystems, especially for monitoring sparse events. This paper presents a review of acoustic wake-up technologies for microsystems. Acoustic sensing, acoustic recognition, and system working mode switching are the basis for constructing acoustic wake-up microsystems. First, state-of-the-art MEMS acoustic transducers suitable for acoustic wake-up microsystems are investigated, including MEMS microphones, MEMS hydrophones, and MEMS acoustic switches. Acoustic transducers with low power consumption, high sensitivity, low noise, and small size are attributes needed by the acoustic wake-up microsystem. Next, acoustic features and acoustic classification algorithms for target and event recognition are studied and summarized. More acoustic features and more computation are generally required to achieve better recognition performance while consuming more power. After that, four different system wake-up architectures are summarized. Acoustic wake-up microsystems with absolutely zero power consumption in sleep mode can be realized in the architecture of zero-power recognition and zero-power sleep. Applications of acoustic wake-up microsystems are then elaborated, which are closely related to scientific research and our daily life. Finally, challenges and future research directions of acoustic wake-up microsystems are elaborated. With breakthroughs in software and hardware technologies, acoustic wake-up microsystems can be deployed for ultra-long-term and ultra-large-scale use in various fields, and play important roles in the Internet of Things.

## 1. Introduction

With the development of the Internet of Things (IoT) and its related technologies, such as the machine learning (ML) algorithm, MEMS transducer, 5G cellular network, etc., a large number of IoT terminals are urgently needed [1]. Microsystems, with the ability of sensing, data processing, transmitting, and executing, are one of the most important terminals of the IoT. In many unattended scenarios, microsystems are used for long-term, large-scale surveillance. However, due to the limited power of the microsystem, the use of low-power electronic components still cannot meet the needs of ultra-long-term surveillance. Energy harvesting can be applied to extend battery life [2]. However, the efficiency of energy harvesting is susceptible to the external environment. Also, the energy harvesting module increases the complexity and size of the microsystem. For many applications in unattended scenarios, events of concern rarely occur. Continuous detection of such sparse events wastes most power of the microsystem [3]. Thus, a wake-up strategy for microsystems is studied. The wake-up strategy refers to the microsystem continuously detecting the events of concern while keeping other modules off, which is also known as low-power sleep mode, and when the events of concern occur, the microsystem turns on all the modules and switches to a high-power active mode. By adopting the wake-up strategy, most of the wasted power is conserved, and the power efficiency is significantly improved, which greatly extends the battery life of the microsystem [4]. Different types of signals are used for event detection in wake-up microsystems, such as acoustic, mechanical, magnetic, optical, infrared, RF, et al. [5,6,7,8,9,10]. Among them, the acoustic signal has the advantages of strong universality, long monitoring distance, rich data information, and abundant acoustic sensors. Therefore, the study of acoustic wake-up microsystems has aroused great interest among researchers.

This review paper presents the technologies for acoustic wake-up microsystems. To achieve acoustic wake-up, microsystems must have the abilities of acoustic sensing, acoustic recognition, and system working mode switching. These are also the key technologies for the acoustic wake-up microsystem. In Section 2, state-of-the-art MEMS acoustic transducers with low power consumption and high sensitivity, which are suitable for acoustic wake-up microsystems, are introduced, including MEMS microphones, MEMS hydrophones, and MEMS acoustic switches. In Section 3, acoustic features capable of event and target recognition are introduced, which are classified into time-domain features, frequency-domain features, and time-frequency domain features. After that, the classification algorithms using the acoustic features as input are investigated, which are divided into the linear classification algorithm and the nonlinear machine learning classification algorithm. In Section 4, according to the power consumption characteristics of the modules in the acoustic wake-up microsystem, four different acoustic wake-up architectures of the microsystem are summarized. In Section 5, applications of the acoustic wake-up microsystem are elaborated, which involve scientific research and our daily use. In Section 6, challenges and future research directions of the acoustic wake-up microsystem are proposed. Section 7 concludes the review.

## 2. MEMS Acoustic Transducer

MEMS acoustic transducers are the hardware basis for acoustic wake-up of microsystems. Here some state-of-the-art MEMS transducers with low power consumption, high sensitivity, and small size, which meet the requirements of acoustic wake-up microsystems, are shown in Table 1.

### 2.1. MEMS Microphone

According to the sensing principle, different types of MEMS microphones are manufactured, including capacitive, piezoelectric, electret, electromagnetic, piezoresistive, and optical microphones. Considering the requirements of low power consumption, high sensitivity, and small size, only capacitive and piezoelectric MEMS microphones are presented, which are also the two most dominant microphone types on the market.

- Capacitive MEMS Microphone

Capacitive MEMS microphones dominate the market with their high signal-noise ratio (SNR) performance and mature manufacturing process [29]. The main structure of the capacitive MEMS microphone is a capacitor made up of a rigid backplate and a flexible diaphragm. A polarization voltage is applied across the capacitor, and acoustic signals are then captured by the flexible diaphragm.

**Figure 1:**
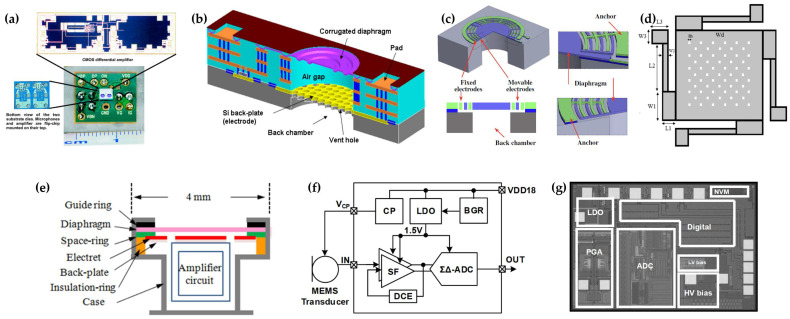
Capacitive MEMS microphones. (**a**) Two MEMS microphones in a differential configuration from Citakovic et al. [11]. (**b**) CMOS MEMS microphone from Huang et al. [12]. (**c**) No-back-plate SOI MEMS microphone from Lo et al. [13]. (**d**) Microphone with Z-shape arms from Ganji et al. [14]. (**e**) Electret capacitive microphone from Woo et al. [15]. (**f**) Microphone based on a triple-sampling delta-sigma ADC from et Lee al. [16]. (**g**) Microphone using differential circuits and internal LDOs from Ceballos et al. [17].

Compared to conventional microphones, MEMS microphones trade off smaller volume with higher noise. By the differential configuration of two MEMS microphones, an SNR of 66 dB is achieved, as shown in Figure 1a [11]. In addition to the high SNR characteristic, a high-sensitivity capacitive CMOS-MEMS microphone is implemented using a standard thin film stacking process, as shown in Figure 1b [12]. The sensitivity of the microphone is 7.9 mV/Pa at 1 kHz with a power consumption of 1.2 mW and a size of 2.34 × 3.2 × 0.865 mm^3^. For capacitive microphones, a back plate is always introduced to form a capacitive structure. However, the back plate brings a damp effect and acoustic impedance which reduce the microphone’s sensitivity, as well as increase its size. A capacitive microphone without a back plate is proposed by replacing the back plate with planar interdigitated sensing electrodes, as shown in Figure 1c [13]. The sensitive part of the microphone has an area of Φ600 μm^2^. To maximize the size advantage of the MEMS microphone, a capacitive microphone with Z-shape arms supported perforated diaphragm is designed, as shown in Figure 1d [14]. The sensitive part of the microphone size is about 0.3 × 0.3 mm^2^, and the sensitivity reaches 2.46 mV/Pa. Another type of capacitive microphone, called the electret capacitive microphone (ECM), has a high sensitivity of up to 100 mV/Pa, as shown in Figure 1e [15]. However, using electret materials increases the difficulty of MEMS processing and its volume. Based on a triple-sampling delta-sigma ADC, a digital capacitive MEMS microphone achieves high sensitivity and low noise performance, and its size is only 0.98 mm^2^, as shown in Figure 1f [16]. Even though the power consumption is reduced to 0.936 mW, it’s still too much for ultra-long-life acoustic wake-up microsystems. Recently, by using differential circuits and internal LDOs, a capacitive microphone with high SNR of 69 dB, small size of 1.13 mm^2^, and low power consumption of 730 μW is achieved, as shown in Figure 1g [17].

- Piezoelectric MEMS Microphone

Piezoelectric MEMS microphones are the second dominant type of MEMS microphone. Compared with the capacitive MEMS microphone, it is less prone to deterioration even after long-term use, and it is less susceptible to moisture and dust due to a gap-free structure. These are essential qualities for ultra-long-life acoustic wake-up microsystems. In addition, lower power consumption or even zero power consumption can be achieved based on the high-sensitivity piezoelectric characteristics.

**Figure 2:**
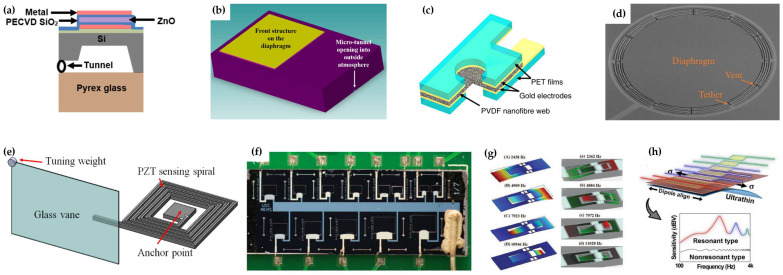
Piezoelectric MEMS microphones. (**a**) Microphone with a ZnO film and a micro-tunnel structure from Prasad et al. [18]. (**b**) High SPL microphone from Ali et al. [19]. (**c**) Microphone based on piezoelectric nanofibers from Lang et al. [20]. (**d**) 430 Hz to 10 kHz resonant microphone from Reger et al. [21]. (**e**) 12.6 V/Pa sensitivity resonant microphone from Pinrod et al. [22]. (**f**) Multi-resonance microphone array from Baumgartel et al. [23]. (**g**) Single structure multi-resonance microphone from Zhang et al. [24]. (**h**) multi-resonance flexible microphone from Wang et al. [25].

A piezoelectric microphone with a ZnO film and a micro-tunnel structure is designed, and a sensitivity of 320.1 μV/Pa is achieved, as shown in Figure 2a [18]. Another ZnO piezoelectric microphone achieves high sound pressure level sensing up to 180 dB, which is available for aeroacoustics applications, as shown in Figure 2b [19], and the sensitivity reaches 130 μV/Pa for broadband from 48 Hz to 54,000 Hz. Unlike common piezoelectric film structures, a high-sensitivity microphone based on piezoelectric nanofibers achieves a sensitivity of 255 mV/Pa, as shown in Figure 2c [20]. To further increase the sensitivity, piezoelectric MEMS microphones based on resonance are investigated. Based on resonance, a high sensitivity of 600 mV/Pa is realized. By designing back cavities with different volumes, the resonant frequency can be adjusted from 430 Hz to 10 kHz, as shown in Figure 2d [21]. By attaching a large glass vane to a MEMS beam, a piezoelectric resonant microphone whose sensitivity is as high as 12.6 V/Pa is achieved, as shown in Figure 2e [22], and the resonant frequency can be as low as 25.2 Hz, which meets the requirement of many surveillance applications. Based on the volt-level output, active electronic amplifiers are no longer required, but the size is about 3.2 × 2.2 × 1 cm^3^. Although resonant microphones have high sensitivity, their narrow resonance bandwidth hinders their application. Multi-frequency resonance is desired to broaden the bandwidth. An array of multiple resonant microphones is designed to widen the frequency band, but the volume increases proportionally, as shown in Figure 2f [23]. Another piezoelectric microphone with multi-frequency resonance without constructing an array is proposed, as shown in Figure 2g [24]. Multi-frequency resonance is achieved by a single structure with multiple vibrational modes. However, the resonant frequencies are all above 2.4 kHz, which is not suitable for common target and event detection. Recently, by mimicking the basilar membrane of the human cochlea, an ultrathin membrane with a tiny asymmetric trapezoidal shape is constructed to enable multi-resonant frequencies with high sensitivity and low noise, as shown in Figure 2h [25].

### 2.2. MEMS Hydrophone

**Figure 3:**
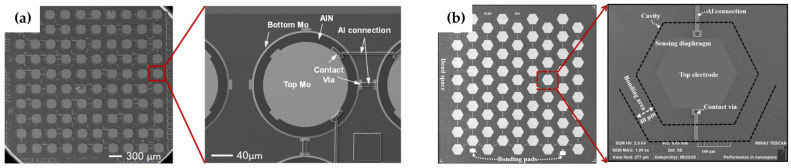
MEMS hydrophones. (**a**) Original circular architecture from Xu et al. [26]. (**b**) Honeycomb architecture from Jia et al. [27].

Some MEMS hydrophones have been reported in recent years, which are used for underwater acoustic sensing. An AlN-based piezoelectric hydrophone is fabricated [30] and further refined [26], as shown in Figure 3a, whose size of the sensing part is 3.5 × 3.5 mm^2^, and overall package size is Φ1.2 × 2.5 cm^3^. Based on the above hydrophone, a biological honeycomb architecture is designed and higher sensitivity and smaller size of the hydrophone are achieved, as shown in Figure 3b [27].

### 2.3. MEMS Acoustic Switch

**Figure 4:**
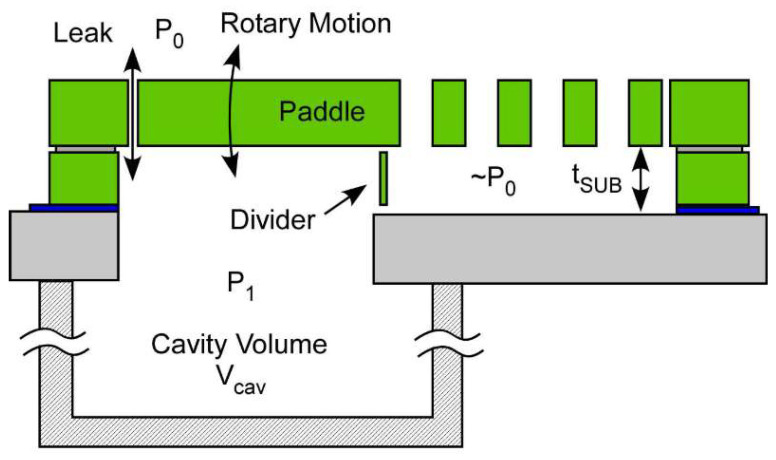
MEMS acoustic switch [28].

MEMS switches are devices that switch conductive contacts on and off. The contacts of MEMS switches are usually distributed on movable cantilever beam structures and thin film structures. There are different ways to actuate the beam and film structures, the common ones being electrostatic force, piezoelectric force, electromagnetic force, and thermal stress. For the acoustic switch, the movable structure is driven by sound pressure. Due to the weak energy in the sound pressure, acoustic switches are rarely reported. A zero-power acoustic switch based on resonance is reported, as shown in Figure 4 [28]. By designing a volume-adjustable cavity structure, the resonance with adjustable frequency is generated which effectively amplifies the sound energy. Micron-scale vibration of the cantilever beam is achieved. However, since the contact is weak, the current-carrying capacity of the switch is only 300 nA, and the switch does not have the ability to remain on.

## 3. Acoustic Recognition

The acoustic recognition process for specific targets or events usually includes data preprocessing, feature extraction, and classification. The data preprocessing is to prepare data for the subsequent feature extraction and classification algorithms, such as data partitioning, filtering, denoising, normalization, DC component removal, and sound mixing. These are conventional analog and digital data processing methods which will not be elaborated further in this paper. Focusing on the acoustic wake-up applications for microsystems, the acoustic features and classification algorithms are discussed in detail.

### 3.1. Acoustic Features

Acoustic features of different categories are discussed in [31,32,33,34]. Since the acoustic features are fundamental to the implementation of acoustic wake-up, they are discussed further in this paper. The features are classified into time domain features and frequency domain features, depending on whether the Fast Fourier transform (FFT) is applied or not, and time-frequency domain features, which are the synthesis of frequency-domain distributions at different times.

#### 3.1.1. Time Domain Features

**Figure 5:**
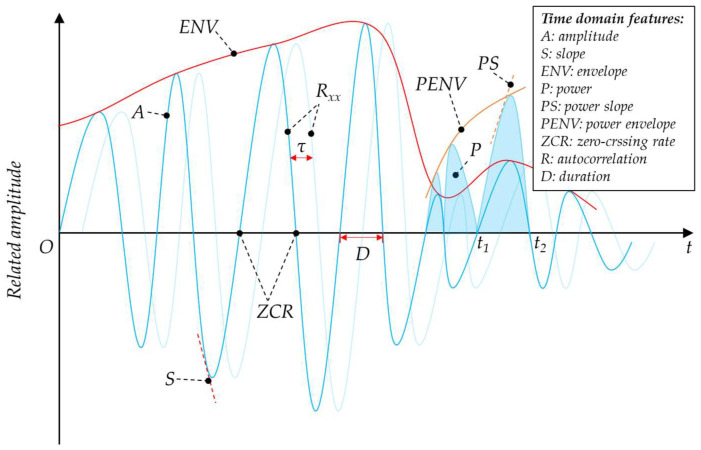
Time domain features.

Time domain features are the most commonly used feature type for acoustic recognition, which can be easily extracted from the acoustic transducers. They are often represented as a graph with time on the abscissa and magnitude-related parameter on the ordinate. For microsystems with limited power and computing resources, easy-to-extracted time domain features are preferred. The commonly used time domain features for acoustic recognition are listed in Figure 5.

- Amplitude

The amplitude (*A*) of the acoustic signal is usually output directly from the analog or digital acoustic sensor. It represents the magnitude of the sound pressure. The amplitudes at different moments further constitute the slope (*S*) and envelope (*ENV*) features. The amplitude, slope, and envelope features characterize the magnitude and variation of the acoustic signal in a simple way.

- Power

Power (*P*) defines the energy in the acoustic signal, which is proportional to the square of the sound pressure. It is often used in the preliminary judgment of the target presence [35]. The average power within a time window is computed as

(1) $$P\left(N\right)=\frac{1}{N}\sum_{n=1}^{N}{\left[x\left(n\right)\omega \left[n\right]\right]}^{2},$$

where $x\left(n\right)$ is the discrete output of the acoustic sensor, and $\omega \left[n\right]$ is a window function of length *N*. Similar to the amplitude feature, the power slope (*PS*) and the power envelope (*PENV*) consist of the sequent power at different instants, which simply suggest the energy variation characteristics.

- Zero-Crossing

The zero-crossing rate (*ZCR*) of an audio frame is the rate of signal sign changes within a time window. It roughly reflects some spectral characteristics in the time domain, and it is easy to be extracted without doing FFT [36].

(2) $$ZCR\left(N\right)=\frac{1}{2}\sum_{i=2}^{N}\left|\mathrm{sgn}\left[x\left(n\right)\right]-\mathrm{sgn}\left[x\left(n-1\right)\right]\right|,$$

where

(3) $$\mathrm{sgn}\left[x\left(n\right)\right]=\left\{\begin{array}{c} \;\;1,\;\;x\left(n\right)\geq 0\\ -1,\;\;x\left(n\right)<0 \end{array}\right..$$

Some other zero crossing-based features are also extracted for acoustic recognition, including zero crossing peak amplitudes (*ZCPA*) and linear prediction zero crossing ratio (*LP-ZCR*) [37].

- Autocorrelation

Autocorrelation (*R*) represents the degree of similarity between two data series that one series is a lagged version of the other, which can represent the resonance characteristics of acoustic signals. For the discrete data, it is given as [38]:

(4) $$R\left(\tau \right)=\sum_{n=1}^{N}x\left(n\right)x\left(n-\tau \right),$$

where *τ* is the number of lags between the 2 data series.

- Duration

Duration (*D*) is the number of samples between two successive real zeros or two successive half-power (also known as 3 dB) points, and it provides information on the fundamental frequency of a waveform [39].

#### 3.1.2. Frequency Domain Features

**Figure 6:**
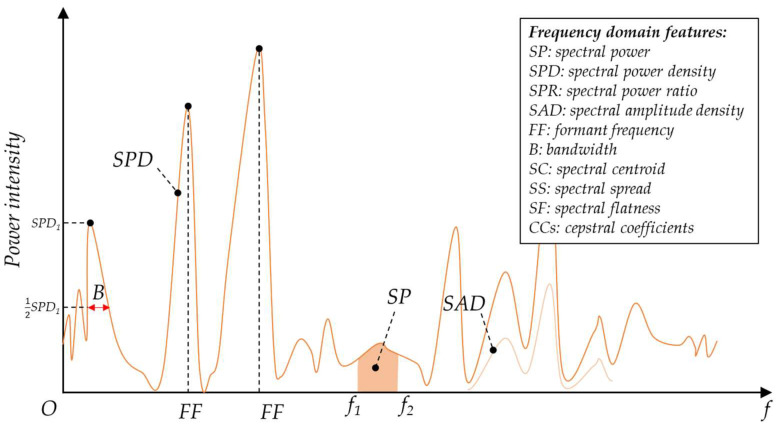
Frequency domain features.

Frequency characteristics are important criteria for acoustic recognition, as different targets and events generate acoustic signals with specific frequency distributions. The FFT deconstructs the acoustic data represented in the time domain into the acoustic data represented in the frequency domain, thereby obtaining the frequency distribution of the acoustic signal. The frequency domain features are often represented as a graph with frequency on the abscissa and spectral density-related parameter on the ordinate. Because of the requirement to use an FFT operation, the computation to obtain frequency-domain features is heavier than that of time-domain features. Nonetheless, the frequency domain features perform much better in acoustic recognition as they are not easily affected by the sound level and the distance of the sound source. Moreover, the differences in frequency domain features of different targets and events are usually more obvious than the differences in time domain features. The commonly used frequency domain features for acoustic recognition are listed in Figure 6.

- Spectral Power

Spectral power density (*SPD*) is a commonly-used metric for target and event recognition, which represents the energy density of different frequency components [40]. Spectral power (*SP*) is obtained by integrating *SPD* along with the frequency. By selecting a specific frequency range, the sub-spectrum power is obtained. To avoid the influence of sound level differences and sensor sensitivity differences on target and event recognition, sub-spectrum power ratio (*SPR*) is used for acoustic recognition. For discrete data, the sub-spectrum power ratio is given as:

(5) $$SPR=\frac{\sum_{f_{i}=f_{1}}^{f_{2}}SPD\left(f_{i}\right)}{\sum_{f}SPD\left(f\right)},$$

where $f_{1}$ and $f_{2}$ are the lower and upper frequencies of a specific sub-band. Spectral amplitude density (*SAD*), which is the square root of the *SPD*, is also mentioned sometimes.

- Formant Frequency

Formant frequencies (*FF*) are the frequencies of the power spectral density extrema. They reflect the main frequency components in the acoustic signal and are useful for distinguishing between different targets and events.

- Bandwidth

Bandwidth (*B*) refers to the frequency range in which the spectral density is above the 3 dB point. It partly reflects the purity of the frequencies in the acoustic signal.

- Spectral Centroid

The spectral centroid (*SC*) is a parameter used to characterize spectral position, which is similar to the mass center of the spectrum. It is calculated as the weighted mean of the frequencies, as follows [41]:

(6) $$SC=\frac{\sum_{k=0}^{N-1}f\left(k\right)SPD\left(f_{k}\right)}{\sum_{k=0}^{N-1}SPD\left(f_{k}\right)}.$$

- Spectral Spread

Spectral spread (*SS*) is the second central moment of the spectrum, which characterizes the extent of the spectrum. The equation is given as [36]:

(7) $$SS=\sqrt{\frac{\sum_{k=0}^{N-1}{\left(k-C\right)}^{2}SPD\left(f_{k}\right)}{\sum_{k=0}^{N-1}SPD\left(f_{k}\right)}}.$$

- Spectral Flatness

Spectral flatness (*SF*), also known as the tonality coefficient, quantifies how similar a sound is to a pure tone. It can be used to identify target signals from white noise-like signals. The equation is [42]

(8) $$SF=\frac{N\cdot \sqrt[{N}]{\prod_{k=0}^{N-1}SPD\left(f_{k}\right)}}{\sum_{k=0}^{N-1}SPD\left(f_{k}\right)}.$$

- Cepstral Coefficient

**Figure 7:**
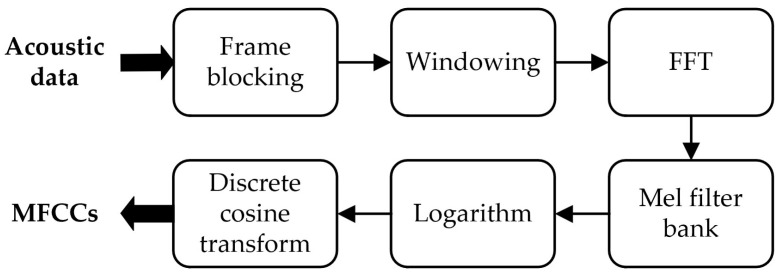
Steps of MFCCs feature extraction.

Cepstral coefficients (*CCs*) are applied for frequency analysis, which involves spectral envelope features. It can be understood as the spectrum of a spectrum in some way. It is reasonable to classify cepstral coefficients as frequency domain features since the FFT operations are performed and they are mainly used for frequency analysis. There are several cepstral coefficients used in acoustic recognition, which are Mel Frequency Cepstral Coefficients (*MFCCs*), Gammatone cepstral coefficients (*GTCCs*), Homomorphic Cepstral Coefficients (*HCCs*), and so on. Among these, *MFCCs* are the most commonly-used ones [43]. *MFCCs* approximate the human auditory system’s response closely, which allows for a better representation of sound characteristics. The steps to get *MFCCs* are shown in Figure 7.

#### 3.1.3. Time-Frequency Domain Features

**Figure 8:**
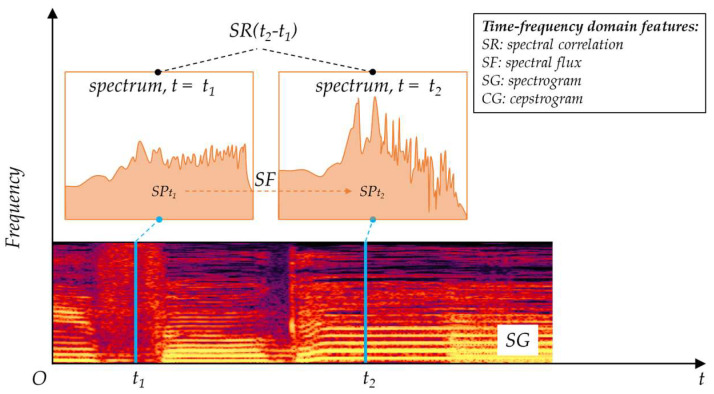
Time-frequency-domain features.

The frequency domain features above are derived from short-term acoustic data. The calculations are based on short-term averages. Thus, the frequency domain features are considered as time-invariant features, as shown in Figure 8. Time-frequency domain features are used for time-varying spectral characteristic analysis. Since richer acoustic information is contained in time-frequency domain features than the time domain features and frequency domain features, better acoustic recognition performance can be achieved, but with a higher computing load [44,45].

- Spectral Correlation

Spectral correlation (*SR*) reflects the periodicity of time-varying frequency features. It is calculated in a similar way to the correlation in time domain signals, which is given as:

(9) $$SR\left(\tau \right)=\sum_{k=1}^{N}SPD_{t_{0}}\left(f_{k}\right)SPD_{t_{0}-\tau }\left(f_{k}\right),$$

where $SPD_{t}$ represents the spectral power density at time *t*.

- Spectral Flux

Spectral flux (*SF*) is the difference in spectral power between two successive acoustic frames. It indicated how fast the acoustic signal changes, which is capable of discriminating different sounds [46].

- Spectrogram

A spectrogram (*SG*) is a representation of the spectrum varying with time, usually depicted as an image with the intensity shown by varying the color or the brightness [47]. Image-processing algorithms can then be applied for spectrogram analysis. Similar to the spectrogram, a cepstrogram (*CG*) is the representation of cepstral coefficients varying with time.

### 3.2. Acoustic Classification Algorithm

Acoustic classification algorithms are executed to distinguish between different targets and events, which use the aforementioned acoustic features as input. Usually, there is more than one target or event of concern. When the number of the concerned targets and events drops to one, the acoustic classification is more often called acoustic detection. In this paper, according to the different mathematical principles of the classification algorithms, the algorithms are divided into linear classification algorithms and nonlinear machine learning classification algorithms.

#### 3.2.1. Linear Classification Algorithm

For the linear classification algorithms, the principle is to calculate the similarity between the features extracted from the acoustic signal and the known target features through linear operations. Next, the category of the target is determined according to the similarity. Most linear classification algorithms are based on or derived from Euclidean distance. The extracted acoustic features form a spatial point in Euclidean space, and each feature corresponds to a coordinate of the spatial point. Thus, *n* features form a spatial point in n-dimensional Euclidean space with coordinates (FTR_1_, FTR_2_, FTR_3_…). The distances from the spatial point to the other known points in the n-dimensional Euclidean space can be derived to quantify the similarity.

- Threshold-Based Method

**Figure 9:**
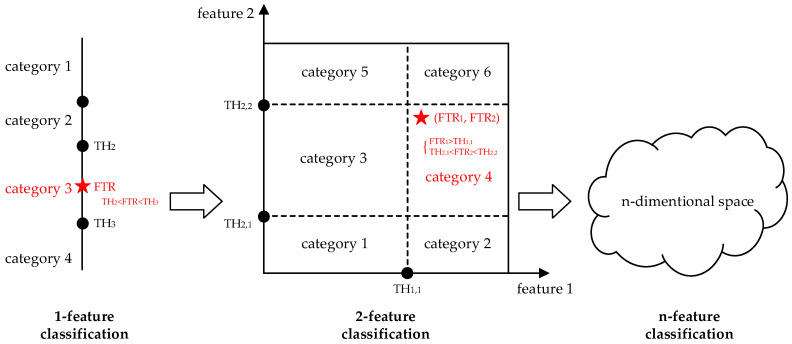
Threshold-based classification.

Threshold (TH)-based classification is one of the simplest classification methods. The category is determined by comparing the extracted features to the known thresholds. For the single-feature classification, the category of the target is determined by the value of the feature, i.e., according to the distance to the known thresholds. Similarly, for the n-feature classification, the category of the target is determined by n distances in n-dimensional space, as shown in Figure 9. To achieve the TH-based acoustic classification, digital or analog comparators are always applied.

- *k*-Nearest Neighbors Method

**Figure 10:**
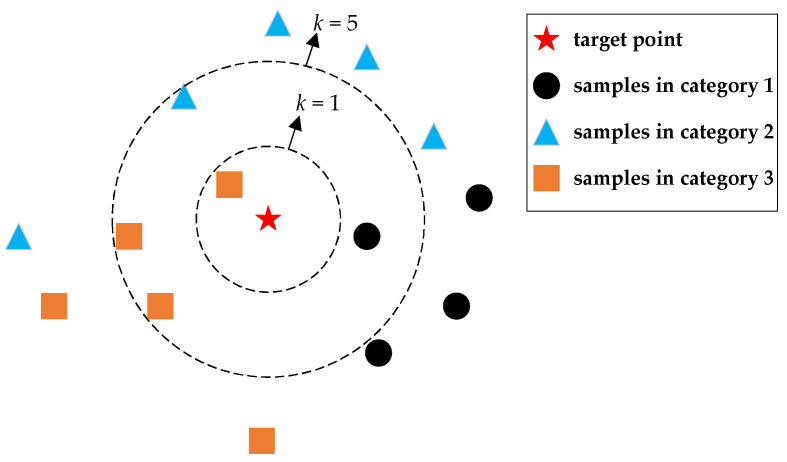
*k*-nearest neighbors classification presented in 2-dimensional form.

The *k*-nearest neighbors (*k*-NN) algorithm implements classification based on the plurality vote by *k* nearest neighbors [48]. As shown in Figure 10, a set of spatial sample points with known coordinates and known categories is established first. After that, calculating the Euclidean distances between the target point and sample points, its *k* nearest neighbors are found. Then, these neighbors vote with the same weight of 1/*k* or with a specific weight based on a weighting rule. The category of the target point is finally determined by the voting result. A microprocessor is required to run the *k*-NN algorithm for acoustic recognition. Since only linear operations are used, a microprocessor with low computing power is sufficient to meet computing needs.

- Nearest Feature Line Method

**Figure 11:**
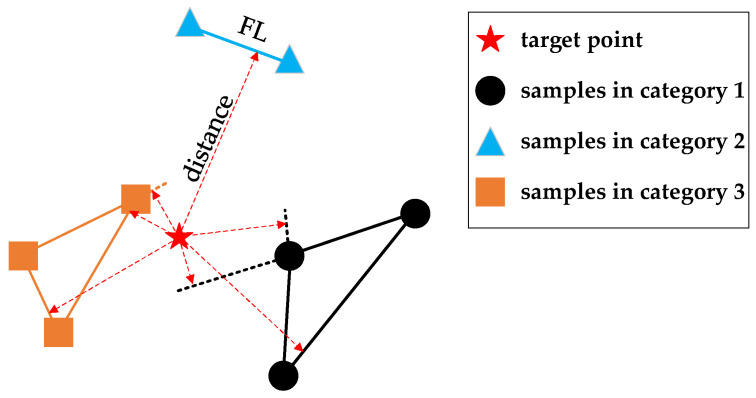
Nearest feature line classification presented in 2-dimensional form.

The nearest feature line (NFL) method is an extension of the *k*-NN, which improves the acoustic classification performance especially when the number of sample points is small [49]. Firstly, a feature line (FL) is defined as a straight line formed by 2 sample points in the same category. Then the distances between the target point and sample points in the *k*-NN method are replaced by the distances between the target point and feature lines in the NFL method, as shown in Figure 11. Since the number of distances in the NFL method is usually larger than in the *k*-NN method, and the calculation of the distance between a point and a line is more complex than between 2 points, the NFL method is more computationally intensive than the *k*-NN method.

#### 3.2.2. Nonlinear Machine Learning Classification Algorithm

Machine learning algorithms play important role in acoustic recognition. Machine learning includes supervised learning and unsupervised learning. The supervised learning is the main method of speech recognition, while some unsupervised machine learning algorithms are also proposed for acoustic recognition [50]. Unsupervised learning requires larger numbers of training samples and more complex training networks, which are not suitable for acoustic wake-up microsystems with low power consumption and low computing power. Until now, only supervised learning has been used for acoustic recognition applications in microsystems. In machine learning classification algorithms, nonlinear models are built by training on feature data instead of building linear models through mathematical analysis. Several machine learning models have been applied for acoustic recognition.

- Support Vector Machine

**Figure 12:**
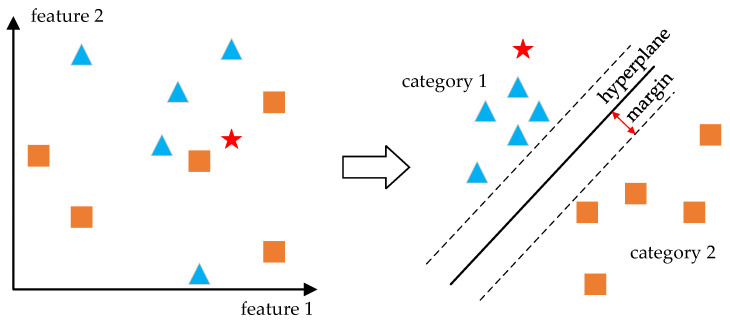
Support vector machine classification presented in 2-dimensional form.

The support vector machine (SVM) is a machine learning model for binary classification, which has been widely used in acoustic recognition due to its good robustness and the appropriate amount of computation [51]. SVM performs classification by mapping the n-dimensional samples to points in m-dimensional space, and a hyperplane is trained to divide the data into 2 categories, as shown in Figure 12. Both linear classification and non-linear classification can be achieved by SVM. To realize the classification of more than 2 types, multiple one-versus-one or one-versus-rest SVM models need to be performed.

- Neural Network

**Figure 13:**
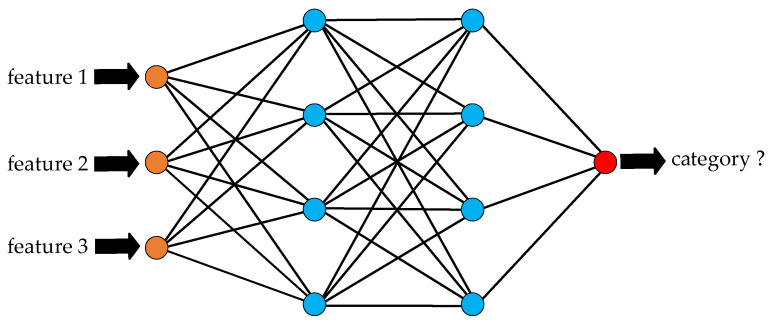
Neural network classification.

Neural network (NN) algorithms perform very well for acoustic-based classification [52,53]. The NN classification algorithms perform non-linear computing based on a collection of connected artificial neurons, as shown in Figure 13. The connections and the strength of the connections are adjusted during the training process. After model training, the acoustic features extracted from the acoustic signal are used as model input, and the category of the acoustic signal will be output from the model.

- Gaussian Mixture Model

**Figure 14:**
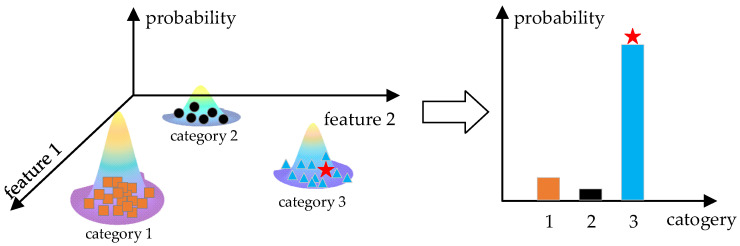
Gaussian mixture model classification presented in 2-dimensional form.

The Gaussian mixture model (GMM) classifies data into different categories based on probability distributions. GMM performs well for acoustic recognition, such as speaker recognition [54]. Firstly, GMM is trained by samples as are the other machine learning algorithms. The target signal is then applied to the GMM to obtain the probabilities of belonging to different categories. Finally, the category of the target signal is determined by the category with the greatest probability, as shown in Figure 14.

- Hidden Markov Model-Based

**Figure 15:**
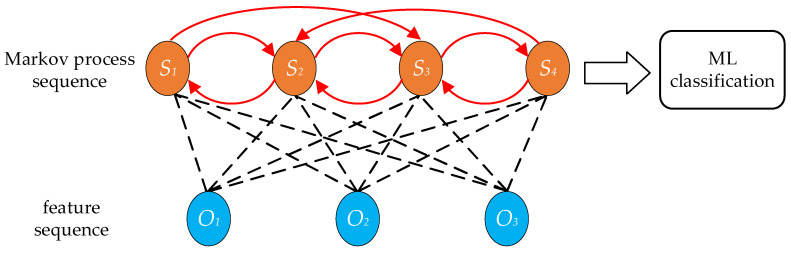
Hidden Markov model-based classification.

The hidden Markov model (HMM) has been used for speech and speaker recognition [55,56]. The time-frequency domain features are usually applied to the HMM as the observable process, and a sequence of hidden Markov processes is constructed. Further acoustic classification is achieved by feeding the sequence into a machine-learning classification algorithm described above, as shown in Figure 15. By applying an HMM-based classification algorithm, the time-frequency features with richer information are used in the classification, thereby improving the classification performance.

Generally, the nonlinear machine learning classification algorithms have higher classification accuracy than the linear classification algorithms, while their computation is heavier, as shown in Table 2. For example, the classification accuracy of the threshold method and *k*-NN method is greatly affected by the extracted features and the chosen samples. Establishing effective sample sets and optimized classification criteria is a tedious process. Although the machine learning classification algorithms do not require strict mathematical analysis and have higher accuracy, their heavy computation is sometimes fatal for microsystems with small size, low power, and long life [57].

For both linear classification algorithms and nonlinear machine learning classification algorithms, the choice of input features needs to be carefully considered. Thus, signal reconstruction algorithms, such as basis pursuit (BP) [58], matching pursuit (MP) [59], and orthogonal matching pursuit (OMP) [60] are often applied to optimize acoustic feature selections.

## 4. System Wake-Up Architecture

Two fundamental modules are required for acoustic wake-up microsystems, which are the wake-up module and the back-end function module. The wake-up module is responsible for acoustic sensing and recognition, and waking up the back-end function module when a specific target appears or a specific event occurs. The back-end function module remains in a low-power or even zero-power sleep mode before waking up, and it performs the main functions of the microsystem after waking up, such as data processing, actuator controlling, and data transceiving. Acoustic wake-up microsystems require ultra-low sleep power consumption and a small size, which results in limited sensing and data processing performance. Although there are many high-performance MEMS acoustic transducers and high-precision classification algorithms applied to the target and event sensing and recognition, not many are able to be implemented in acoustic wake-up microsystems. In this section, system wake-up architectures of the acoustic microsystem are introduced, as shown in Table 3. The system wake-up architectures are divided into four categories according to whether the wake-up module or the back-end function module consumes power in sleep mode. The power consumption caused by the current leakage of electronic devices, batteries, etc., is treated as zero power consumption. Some acoustic wake-up chips, which have not been used but are capable of the construction of an entire microsystem, are also reported.

### 4.1. Architecture 1: Low-Power Recognition and Low-Power Sleep

**Figure 16:**
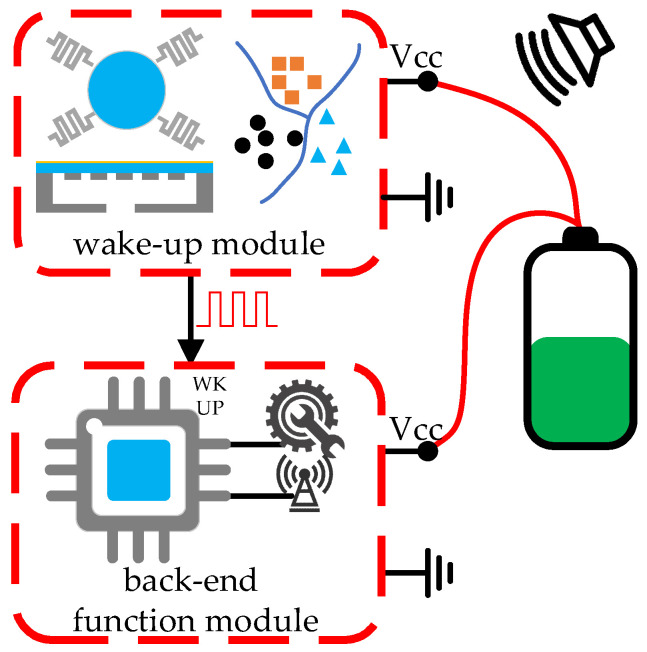
Architecture 1: low-power recognition and low-power sleep.

In the low-power recognition and low-power sleep architecture, aka Architecture 1 in this paper, when the microsystem is in sleep mode, the wake-up module consumes power for acoustic sensing and recognition, while the back-end function module also consumes power waiting for the wake-up signal, usually a voltage signal of high or low, from the wake-up module, as shown in Figure 16. In the back-end function module, there must be a chip capable of switching between high-power active mode and low-power sleep mode. This architecture is the most used and most mature wake-up architecture in various electronic devices, and also in microsystems.

**Figure 17:**
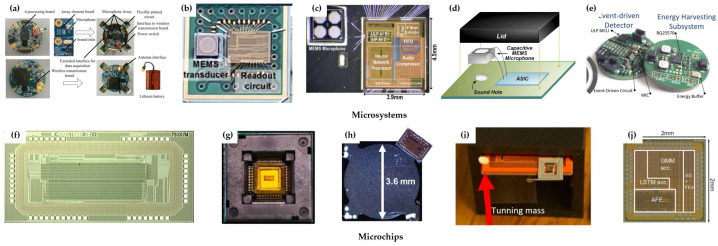
Acoustic wake-up microsystems and microchips in Architecture 1. (**a**–**e**) Microsystems. (**f**–**j**) Microchips. [22,61,62,63,64,65,66,67,68,69].

An acoustic wake-up microsystem in this architecture is reported, which achieves target detecting, classifying, and tracking in the real wild area, as shown in Figure 17a [61]. The microsystem consumes 13.8 mW in sleep mode and has a long-term continuous monitoring capability of about 33 days. The whole weight including the battery is 145 g and the volume is 1056 cm^3^, which is a bit bulky for the microsystem. A simple acoustic wake-up microsystem with μW-level power consumption is then reported, which is made up of a MEMS microphone and a readout circuit, as shown in Figure 17b [62]. When an acoustic event within the specific voice band occurs, the system wakes up and begins to output the acoustic data sensed by the microphone. Then, a mixer-based circuit and a low-power NN algorithm are applied to a microsystem to achieve acoustic recognition with nW-level power consumption, as shown in Figure 17c [63]. Both speech and non-speech detections are realized, with a power consumption of 142 nW. When a target event is detected, the system is activated to a high-performance mode. Among all the acoustic wake-up microsystems with Architecture 1, a 12 nW microsystem is the one with the lowest power consumption, as shown in Figure 17d [64]. By optimizing the power consumption of algorithm-circuit and electronic components, the microsystem realizes acoustic event identification with 12 nW consumption. In addition to the applications on land, there is also a report for the underwater application. An acoustic wake-up microsystem containing a hydrophone for underwater deployment is achieved, as shown in Figure 17e [65]. A machine-learning algorithm runs on an onboard microcontroller, and different acoustic signals are classified with an accuracy of up to 95.89%.

Some low-power acoustic wake-up microchips without back-end function modules are reported, too. A 305.5 μW wake-up chip, 300 μW for the MEMS microphone and 5.5 μW for the signal classification circuit, is reported for acoustic recognition of the tracked vehicle and wheeled vehicle, as shown in Figure 17f [66]. A distance of more than 500 m has been achieved for heavy tracked vehicle recognition. A 75 nW wake-up chip is reported to detect heart rate, epilepsy, and keyword, which can be further applied to acoustic wake-up microsystems for practical use, as shown in Figure 17g [67]. A wake-up chip for ultrasonic signal detection is reported with a smaller size of 14.5 mm^2^, as shown in Figure 17h [68]. Its power consumption reduces to 8 nW, which is comparable to the leakage power of current batteries. By applying a zero-power MEMS microphone, a wake-up chip with power consumption as low as 6 nW is achieved, which is shown in Figure 17i [22]. By adjusting the resonant frequency of the zero-power microphone, the acoustic signal with a specified frequency is successfully detected, including the signal from the generator and the truck. However, it only detects one target in one setting. The resonant frequency of the microphone needs to be tuned by tunning weight. The acoustic wake-up chips above classify the target all by the threshold-based method, which is the simplest classification algorithm with low accuracy. A wake-up chip for keyword spotting and speaker verification using GMM and NN classification algorithms is reported, while the power consumption is up to 10 μW, as shown in Figure 17j [69].

### 4.2. Architecture 2: Zero-Power Recognition and Low-Power Sleep

**Figure 18:**
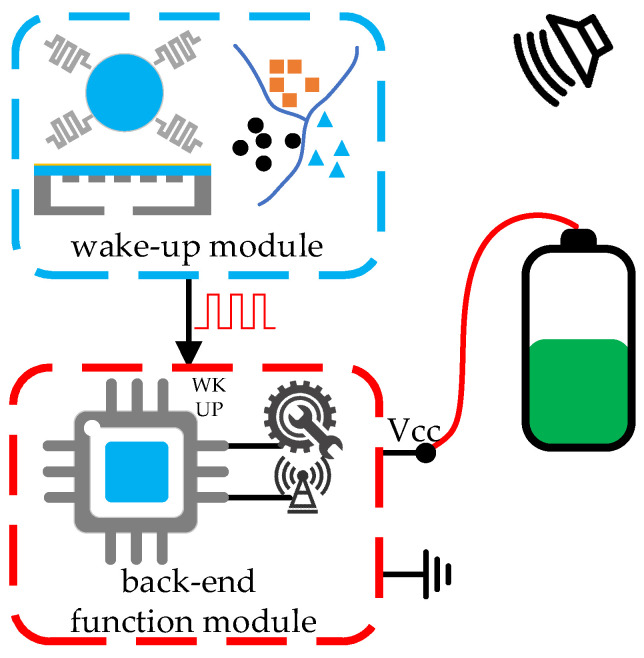
Architecture 2: zero-power recognition and low-power sleep.

In the zero-power recognition and low-power sleep architecture, aka Architecture 2, the wake-up module performs acoustic sensing and recognition with zero power consumption, while the back-end function module remains the same as in Architecture 1, as shown in Figure 18. Zero-power sensing and data processing technologies, such as high-sensitivity piezoelectric transducers, passive amplifiers, passive filters, and passive classifiers, are required. When the target acoustic signal appears, the wake-up module recognizes it and then generates a wake-up signal for the back-end function module.

**Figure 19:**
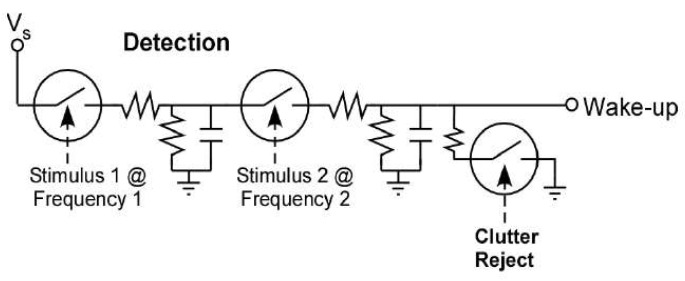
Acoustic wake-up microchip in Architecture 2 [28].

A zero-power wake-up chip made up of the acoustic switch in [28] has been used for generator and truck detection as shown in Figure 19. Three acoustic resonant switches with different resonant frequencies are used as passive filters for target detection and noise cancellation. The power consumption caused by the current leakage in the chip is less than 1 nW.

### 4.3. Architecture 3: Low-Power Recognition and Zero-Power Sleep

**Figure 20:**
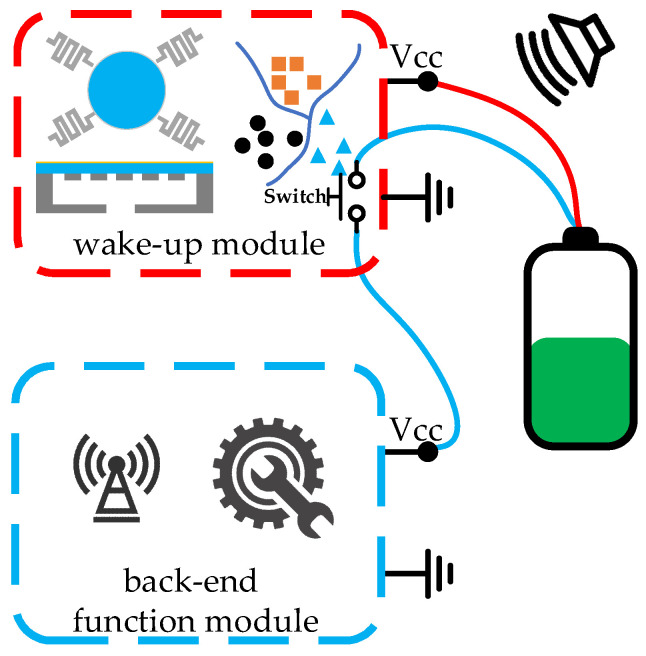
Architecture 3: low-power recognition and zero-power sleep.

In the low-power recognition and zero-power sleep architecture, aka Architecture 3, the wake-up module performs acoustic sensing and recognition with power consumption, which is similar to the wake-up module in Architecture 1. However, there is a switch in the module, which is used for controlling the current flowing through the back-end functional module, as shown in Figure 20. In addition, a chip with the function of switching working modes in the back-end function module is no longer needed. In sleep mode, the back-end function module is powered off instead of in a low-power sleep state. This switch-included wake-up module is much more universal and can easily be used to reform the wake-up function of various electronic systems. Nonetheless, the switch increases the size and power consumption of the wake-up module.

**Figure 21:**
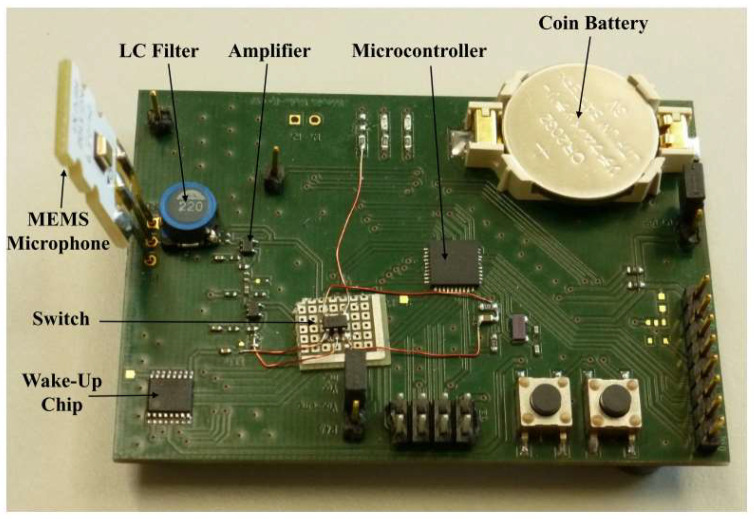
Acoustic wake-up microchip in Architecture 3 [70].

A wake-up chip containing a switch is able to turn off the backend function module completely instead of keeping it in a low-power sleep mode, as shown in Figure 21 [70]. It should be noted that the wake-up chip in Figure 21 is different from the definition in this paper. Instead, the entire module in Figure 21 is regarded as the wake-up chip since it only achieves functions of acoustic sensing, recognizing, and wake-up. The power consumption of the chip is 420 μW, and the size is of centimeter-level. Optimizations of the chip are required for its further application in acoustic wake-up microsystems.

### 4.4. Architecture 4: Zero-Power Recognition and Zero-Power Sleep

**Figure 22:**
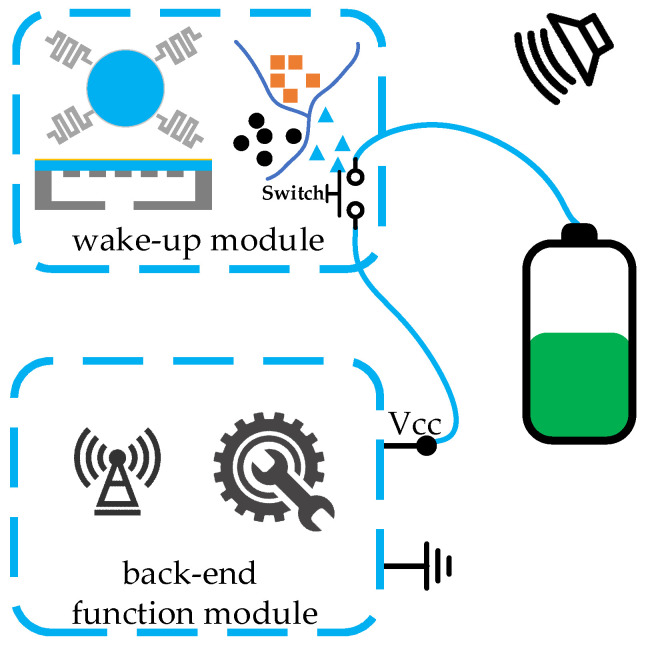
Architecture 4: zero-power recognition and zero-power sleep.

In the zero-power recognition and zero-power sleep architecture, aka Architecture 4, the microsystem consumes absolutely zero power in sleep mode. A wake-up module with zero-power sensing, recognition, and circuit switching is the key to this architecture, as shown in Figure 22. Acoustic sensing, signal processing, and switch actuation are all powered by the energy in the acoustic signal.

**Figure 23:**
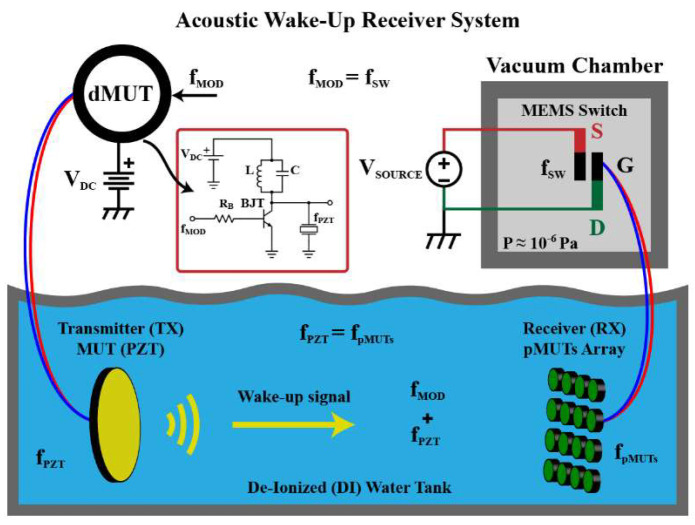
Acoustic wake-up microsystem in Architecture 4 [72].

A zero-power acoustic wake-up receiver, made up of an ultrasonic microphone array and a MEMS electrostatic switch is shown in Figure 23 [72]. When receiving target ultrasonic data, the zero-power piezoelectric microphone array generates a voltage to drive the biased MEMS electrostatic switch. Thus, zero-power consumption for ultrasonic data reception is achieved. Due to the low current-carrying capacity of the MEMS electrostatic switch in the receiver, the receiver can only generate a wake-up signal but not directly turn on a backend function module. Thus, the output voltage from the receiver is further induced into a CMOS load switch [71]. When the target signal appears, the CMOS load switch is driven on, and the backend function module, which is an implanted medical device, is powered on and wakes up.

## 5. Applications

Acoustic wake-up microsystems have the characteristics of low power consumption, small size, and long battery life, which lead to large-scale and long-term acoustic monitoring. The wake-up technology significantly improves energy efficiency and battery life, especially for the detection of rare events [3]. In this paper, the applications the acoustic wake-up microsystems can be used are summarized. Some of the applications have been already implemented, while others are expected to be implemented in the future.

### 5.1. Perimeter Surveillance

For vast border areas, wilderness areas, scattered warehouses, etc., detecting intrusions, although rarely happening, is very important for security reasons. Targets such as human beings, vehicles, and wildlife, are of constant concern for both civilian and military use [39,66,73,74,75,76]. Traditional high-power monitoring methods, such as live cameras, require a power grid for power supply which is impractical for many applications. The presence and movement of specific targets are always accompanied by sounds with specific acoustic features. Thus, targets can be detected and recognized by applying an acoustic wake-up microsystem. When multiple microsystems are applied to form a sensing network, moving target localization and tracking can also be achieved by analyzing the amplitude differences, time of arrival (TOA), and time difference of arrival (TDOA) of the acoustic signals [77,78,79].

### 5.2. Structure Health Monitoring

Structural health monitoring of important infrastructures, such as bridges, dams, tunnels, and transmission towers, is related to our safety. Timely detection of abnormalities and failures of their structure is urgently desired to avoid heavy losses. When cracks appear in a structure, its acoustic signature changes. Thus, structure health monitoring can be done by acoustic recognition [80]. Most structure health monitoring requires active acoustic emission with high power consumption [81], which is not suitable for the acoustic wake-up microsystem. Fortunately, passive acoustic emissions may be utilized for structure health monitoring without power consumption, such as the sounds produced by the cars on the bridges, and by the running water through the dams and tunnels. By deploying acoustic wake-up microsystems on these infrastructures, low-power-consumption, long-term, and real-time monitoring of structural abnormalities can be achieved, which will guarantee the safety of people and property.

### 5.3. Human Health Monitoring

Human health has always been the most important issue in our daily lives. Medical diagnoses by wearable acoustic monitoring devices have been investigated, including heart and lung sound recognition, and wheeze detection [82,83,84]. In the foreseeable future, more acoustic microsystems will be applied to the continuous monitoring of abnormal health signals to ensure early detection and treatment. With the acoustic wake-up technology, ultra-long-term monitoring without charging or battery replacement can be realized, which greatly improves the convenience of the use of wearable health monitoring devices.

### 5.4. Agriculture Application

Agriculture is the practice of plant and livestock cultivation. It has been the foundation of our lives since ancient times. The application of modern technologies in agriculture can effectively increase the production of crops and livestock, releasing farmers and herdsmen from heavy work. Weather conditions [85,86], insects [87,88], birds [89], and livestock behaviors [90], which are closely related to agricultural production, can be detected by acoustic signals. Acoustic wake-up microsystems are worthy of application in these instances, especially for rare exceptions, such as severe weather conditions, invasive alien species, and unknown avian influenza infections, which occur rarely but impact significantly.

### 5.5. Biodiversity Research

Biodiversity research is important for ecological stability and life science research. Finding different creatures, especially rare ones, in the vast wilderness or the deep sea is sometimes difficult. Bioacoustics signals can be used for biodiversity studies both on land and underwater [91,92,93,94,95,96]. A vast, low-power, long-life monitoring network can be built by the acoustic wake-up microsystem to achieve biodiversity research. Only useful acoustic signals are detected and processed, which greatly reduces the amount of useless information.

### 5.6. Smart City

Urban life is full of various acoustic signals, which makes the ears so important to us. Acoustic wake-up microsystems are like the ears of a smart city that are used for monitoring various events and targets. Acoustic signals are already investigated for indoor moving target detection [97,98], traffic control [99], speaker recognition [100], and providing human interfaces to IoT ends [101]. With the increasing number of acoustic microsystems, a wider and more powerful IoT will greatly facilitate our daily lives.

## 6. Challenges and Future Research Directions

The core purpose of the acoustic wake-up microsystem is to significantly extend the battery life for sparse acoustic event detection, by means of saving wasted power, improving power efficiency, and reducing power consumption. But it also brings some disadvantages. Under the condition of strictly limiting the sleep power consumption of the microsystem, its acoustic recognition ability is reduced, including limited identifiable sound categories, limited recognition sensitivity, and limited recognition accuracy. Until now, the number of acoustic wake-up microsystems is still small, especially systems with Architecture 2, Architecture 3, or Architecture 4. Microsystem technology is a system technology including hardware and software. To better promote the development of the acoustic wake-up microsystem, it is necessary to conduct research on both software and hardware, which is aimed at lower sleep power consumption and higher recognition capabilities.

### 6.1. Software

Software in a microsystem must be efficient and designed for specific applications. Due to the limited power supply and long life requirement of the acoustic wake-up microsystem, the software is always optimized to reduce computation and improve efficiency, including data input, output, and calculation processes. For the acoustic wake-up microsystem, the acoustic classification algorithm is the core of the software. Algorithms with higher classification accuracy and lower computation amount are desired. Thus, research on acoustic feature selection and extraction, and feature-based classification algorithm needs to be further studied according to the microsystem’s application scenarios and requirements.

### 6.2. Hardware

For the hardware, nanowatt and zero-power components are required for the acoustic wake-up microsystem. For acoustic sensing, the technology of MEMS acoustic transducers needs to be studied to improve their uses, including the MEMS microphone, MEMS hydrophone, and MEMS acoustic switch, and to improve their performance, including higher sensitivity, lower power or even zero power consumption, lower noise and smaller size. A high sensitivity piezoelectric microphone can lower the power consumption, and the voltage output from the microphone may directly drive a MEMS switch or a CMOS switch without using an active amplifier. For acoustic signal processing, nanowatt processors are needed to implement machine learning algorithms and other classification algorithms. Other low-power or even zero-power signal processing components in the system circuit are also required, such as the amplifier, analog-to-digital (ADC) converter, solid-state relay, clock, etc. The current leakage in the circuit components is non-negligible in ultra-long-life wake-up microsystem applications. To implement acoustic wake-up microsystems of Architecture 3 and Architecture 4, a switch with little current leakage is essential. The CMOS switch with ultra-low current leakage, MEMS electrostatic switch with low trigger threshold, and zero-power acoustic switch with wider bandwidth can be tested as solutions. Especially for Architecture 4, there is an urgent need for a zero-power acoustic switch that can respond to multiple frequency bands and remain on without consuming power.

## 7. Conclusions

Acoustic sensing, acoustic recognition, and system working modes switching are the basic functions and core technologies of acoustic wake-up microsystems. In this paper, low-power and high-sensitivity MEMS acoustic transducers, linear and nonlinear acoustic recognition algorithms, and state-of-the-art acoustic wake-up microsystems with different wake-up architectures are presented. For long-life acoustic wake-up microsystems, low-power or even zero-power MEMS acoustic transducers are required. With the development of MEMS acoustic transducers, more and more MEMS microphones, MEMS hydrophones, and MEMS acoustic switches with low power consumption, high sensitivity, low noise, and small size, are reported. By applying them to microsystems, acoustic wake-up with higher accuracy and lower power consumption can be achieved. As for acoustic recognition, specific acoustic features need to be extracted and applied to classification algorithms. The selection of acoustic features and classification algorithms needs to be considered according to the power consumption, transducer performance, and microprocessor performance of the microsystem. Combining state-of-the-art acoustic recognition algorithms with the acoustic signal sensing and processing modules enables system wake-up architectures of ultra-lower power consumption, or even absolutely zero power consumption. With the advancement of software and hardware technology, numerous acoustic wake-up microsystems with smaller sizes, higher energy efficiency, longer battery life, and higher intelligence will be developed and applied in various fields of IoT.

## Figures and Tables

**Table 1:** MEMS acoustic transducers.

Type	Principle	Main Structure and Material	Power Consumption	Size	Frequency Range (Hz)	Resonant Frequency (Hz)	Sensitivity	SNR	Year	Ref.
MEMS microphone	Capacitive	Compliant membrane	-	2.6 × 3.2 × 0.865 mm^3^	20–20,000	24.15 k	-	65.6 dB	2009	[11]
	Capacitive	Corrugated diaphragm	1.2 mW	2.35 × 1.65 × 1.2 mm^3^	100–10,000	-	7.9 mV/Pa	55 dB	2011	[12]
	Capacitive	Planar interdigitated	-	Φ600 μm^2^	1000–20,000	-	0.99 mV/Pa	-	2015	[13]
	Capacitive	Perforated diaphragm	-	0.3 × 0.3 mm^2^	1–20,000	60 k	2.46 mV/Pa	-	2018	[14]
	Capacitive	Graphene−PMMA diaphragm	-	Φ4 × 3.2 mm^3^	0–10,000	7 k	100 mV/Pa	20 dB	2017	[15]
	Capacitive	Triple-sampling ADC	0.936 mW	0.98 mm^2^	20–20,000	-	38.0 mV/Pa	62.1 dBA	2022	[16]
	Capacitive	Differential circuits	730 μW	1.13 mm^2^	-	-	-	69 dBA	2022	[17]
	Piezoelectric	ZnO film	-	3 × 3 mm^2^	30–8000	42.875 k	320.1 μV/Pa	-	2021	[18]
	Piezoelectric	ZnO film	-	1.5 × 1.5 mm^2^	48–54,000	99.6 k	130 μV/Pa	-	2022	[19]
	Piezoelectric	Piezoelectric nanofiber	-	-	400–1500	-	266 mV/Pa	-	2016	[20]
	Piezoelectric	AlN diagram	0	-	-	0.43 k–10 k	600 mV/Pa	-	2017	[21]
	Piezoelectric	PZT spiral	0	3.2 × 2.2 × 1 cm^3^	-	>25.2	12.6 V/Pa	-	2018	[22]
	Piezoelectric	ZnO film	-	4 × 11 mm^2^	240–6500	0.86 k–6.263 k	2.5–202.6 mV/Pa	-	2012	[23]
	Piezoelectric	AlN cantilevers	-	5.5 × 5.5 mm^2^	-	2.4 k, 4.9 k, 8.0 k, 11.0 k	19.7 mV/Pa	-	2016	[24]
	Piezoelectric	PZT membrane	-	1 × 2.5 cm^2^	-	0.1 k–4 k	103 mV/Pa	92 dB	2021	[25]
MEMS hydrophone	Piezoelectric	AlN film	-	Φ1.2 × 2.5 cm^3^	10–8000	-	1 μV/Pa	60 dB	2018	[26]
	Piezoelectric	AlN film	4.5 mW	1.5 × 0.8 × 2 cm^3^	10–50,000		1.26 μV/Pa	58.7 dB	2021	[27]
MEMS acoustic switch	Resonant	Rotational Paddle	0	≤15 cm^3^	-	62.7–80	0.005 Pa (threshold)	-	2018	[28]

**Table 2:** Acoustic classification algorithm.

Type	Classifier	Computation	Accuracy
Linear classification	Threshold-based	★	★
	*k*-NN	★☆	★☆
	NFL	★★	★★
Nonlinear machine learning classification	SVM	★★★	★★★
	NN	★★★★	★★★★
	GMM	★★★☆	★★★☆
	HMM-based	★★★★☆	★★★★☆

* ★ and ☆ indicate the amount of computation and the level of accuracy, and more stars indicate greater computation and higher accuracy; ☆ represent half ★.

**Table 3:** Acoustic wake-up microsystems.

System Architecture	Acoustic Recognition		Target	Size	Sleep Power Consumption			Accuracy	False Alarm	Year	Ref.
	**Feature**	**Classifier**			Wake-Up Module	Back-End Module	Total				
Architecture 1	Spectral correlation	Threshold and GMM	Truck, wheeled vehicle, tracked vehicle	Φ50 × 130 mm^3^	-	-	13.8 mW	92.6%	<5%	2019	[61]
	Amplitude envelope	Threshold	Voice-band	-	8.25 μW	44.55 μW	52.8 μW	-	-	2020	[62]
	Sub-spectrum amplitude	NN	Speech/non-speech	4.5 × 3.9 mm^2^	66 nW	76 nW	142 nW	>90%	-	2019	[63]
	Spectrum amplitude, average power	SVM	Generator, truck, car	2.15 × 1.6 mm^2^	-	-	12 nW	>95%	-	2017	[64]
	Spectrogram	ML	Submarine, ship, rain, surface ice	-	26.89 μW	35.11 μW	62 μW	95.89%	-	2019	[65]
	Autocorrelation	Threshold	Wheeled vehicle, tracked vehicle	3 × 1.5 mm^2^	305.5 μW	-	-	-	-	2004 *	[66]
	Amplitude, slope	Threshold	Heart rate, epilepsy, keyword	-	75 nW	-	-	-	-	2021 *	[67]
	Envelope	Threshold	Ultrasonic signal	14.5 mm^2^	8 nW	-	-	-	-	2019 *	[68]
	Sub-spectrum energy	Threshold	Generator, truck	3.2 × 2.2 × 1 cm^3^	6 nW	-	-	100%	1/h	2018 *	[22]
	Power, MFCCs	GMM, NN	Keyword spotting	2 × 2 mm^2^	10.6 μW	-	-	>94%	-	2020 *	[69]
Architecture 2	Sub-spectrum energy	Threshold	Generator, truck	-	<1 nW	-	-	100%	0	2018 *	[28]
Architecture 3	Sub-spectrum energy	Threshold	Ultrasonic signal	-	420 μW	0	420 μW	-	-	2016 *	[70]
Architecture 4	Sub-spectrum energy	Threshold	Fixed frequency ultrasound	-	0	<10 nW	<10 nW	-	-	2022	[71]

* An acoustic wake-up chip, not a complete acoustic wake-up microsystem.

## Data Availability

Not applicable.
